# Erythrocyte Salt Sedimentation Assay Does Not Predict Response to Renal Denervation

**DOI:** 10.3389/fmed.2018.00051

**Published:** 2018-03-09

**Authors:** Oliver Vonend, Ole Martin, Lars C. Rump, Patrick Kroepil, Johannes Stegbauer

**Affiliations:** ^1^Nierenzentrum, DKD Helios Klinik Wiesbaden, Wiesbaden, Germany; ^2^Department of Nephrology, Medical Faculty, University Hospital Düsseldorf, Heinrich-Heine-University Düsseldorf, Düsseldorf, Germany; ^3^Departement of Diagnostic and Interventional Radiology, Medical Faculty, University Hospital Düsseldorf, Heinrich-Heine-University Düsseldorf, Düsseldorf, Germany

**Keywords:** hypertension, renal denevation, salt sensitivity of blood pressure, salt sensitivity, blood pressure

## Abstract

Renal denervation (RDN) has recently been shown to be effective in patients without antihypertensive medication. However, about 30% of patients do not respond to RDN, and therefore, there exists a need to find predictors of response. Individuals are either salt-sensitive (SS) or non-salt-sensitive (NSS) in terms of their blood pressure (BP) regulation. The sympathetic nervous system can influence water and salt handling. RDN reduces sympathetic drive and has an impact on salt excretion. The present study was conducted to test the influence of salt sensitivity in terms of the BP reducing effect after RDN procedure. Salt sensitivity was estimated using the *in vitro* Erythrocyte Salt Sedimentation Assay (ESS). In 88 patients with resistant hypertension, RDN was performed. Office BP and lab testing were performed at baseline and at month 1, 3, 6, 12, 18, and 24 after RDN. A responder rate of 64.7% has been observed. Salt sensitivity measurements (ESS-Test) were completed in a subgroup of 37 patients with resistant hypertension. In this group, 15 were SS and 17 were salt-resistant according to the *in vitro* assay, respectively. The responder rate was 60% in SS patients and 59.1% in NSS patients, respectively. Electrolytes as well as aldosterone and renin levels did not differ between the two groups at baseline and in the follow-up measurements. The present study showed that salt sensitivity, estimated using the ESS *in vitro* test, did not affect the outcome of RDN and, therefore, does not help to identify patients suitable for RDN.

## Introduction

The important role of sodium in the development and maintenance of hypertension has been shown in numerous animal models and human studies (1). The sympathetic nervous system plays a pivotal role in sodium handling since sympathetic nerves can contribute to the physiology of salt-sensitive (SS) hypertension (2, 3). After an impressive start, renal denervation (RDN) struggled to show its effectiveness in the HTN-3 trial (4). The HTN-3 trial was the first blinded, randomized, and in particularly sham-controlled trial analyzing the effectiveness and safety of RDN. The office BP was lowered substantially by −14 ± 24 mmHg after the RDN procedure. However, RDN failed to show its superiority to sham intervention since the drop of office BP was similarly in the control group without RDN (sham group −12 ± 26 mmhg). There were no major safety concerns reported in both groups. Several explanations were given to interpret the inhomogeneous results (5). *Post hoc* evaluation suggested adherence problems, insufficient RDN, inclusion of patients with isolated systolic hypertension, and other factors. Some of these aspects have been addressed in the latest SPYRAL HTN-OFF MED trial design (6). Even before the HTN-3 trial was carried out, it was assumed that the office blood pressure (BP) reduction of the HTN-1 and -2 trials were overestimated due to a lack of double-blinding (7).

In the recent SPYRAL HTN-OFF MED study it has been shown, that RDN effectively reduces blood pressure in the absence of antihypertensive drugs in patients with an office systolic BP between 150 and 180 mmHg in the absence of antihypertensive drugs (6). However, approximately one-third of patients did not respond to RDN in that trial.

Despite the above mentioned factors, not many valid parameters have been identified till now, which allow to predtict response to RDN. In addition, the mechanisms how RDN reduces BP in man are not fully understood. Measuring an increased sympathetic activity at baseline (e.g. by norepinephrine spillover or muscle sympathetic nerve activity) would certainly be the gold standard to select patients with sympathetic overactivity for RDN (8). However, the methods described can hardly be integrated in the clinical routine. Therefore, it seems essential to find other easy-to-use predictors for a response to RDN. Evaluation of the salt sensitivity using the salt blood test (SBT) would be such an assay.

Recently, it has been shown in patients with resistant hypertension, that RDN might influence sodium excretion rate (9). In addition, patients with resistant hypertension are often characterized by an increased salt sensitivity (10).

In order to identify parameters that might predict BP response to RDN therapy, the influence of salt sensitivity were analyzed using an *in vitro* assay in a subgroup of 37 patients with resistant hypertension and RDN. This now commercially available assay could easily be integrated in the clinical workup before planning RDN. As described by the developer of the SBT, the erythrocyte sodium sensitivity (ESS), and the red blood cells (RBC) sodium buffer capacity does correspond to the patients’ individual salt sensitivity (11, 12).

## Materials and Methods

In the years 2010–2015, *n* = 88 patients underwent RDN in a single center and were included for the present investigation. The patients received RDN in a specialized center (University Duesseldorf) to treat resistant hypertension. The patients were part of a local register (Ethic No. 3848) and/or included in the “GREAT Register” (13). The institutional review board of the Medical Faculty University of Düsseldorf approved this prospective study (NR 3848). A written informed consent was obtained from all participants of this study.

Only patients with long-lasting history of resistant hypertension were included. According to the current definition of resistant hypertension, patients with an office BP > 140/90 mmHg with at least three antihypertensive drugs at maximal tolerated doses, of which one is diuretic were further evaluated for RDN. Before inclusion into the study, medical history including DM, cardiovascular risk factors, and OSAS were evaluated and secondary hypertension forms were excluded and medication was optimized according to the guidelines (14). RDN was performed as described elsewhere using the Medtronic Symplicity single electrode catheter (4, 15). The patients were evaluated at baseline prior RDN and 1, 3, 6, 12, 18, and 24 months after the procedure. Office BP (automated measurement, mean of three readings), ABPM and lab testing were performed at baseline and during follow-up. The blood test included hemoglobin, electrolytes, serum-creatinine (eGFR CKD-EPI), BUN, cystatin C, metanephrines, aldosterone, and renin. Urine sodium, potassium, urine-creatinine, and albumin were also measured.

Data (office BP and Lab values) of all 88 patients were available at baseline and at the 6 months follow-up; whereas only half of the patients had ABPM measurements due to lack of compliance. The number of available patient data at follow-up month 12, 18, and 24 were 76, 64, and 64, respectively.

The presence of low or high salt sensitivity was prospectively tested in a subgroup of *n* = 37 patients (years 2014–2015) using a recently established SBT before RDN therapy (11). In brief, red blood cells were suspended in stabilized solutions with different sodium concentrations. These ready to use solutions (SBT-KIT) were kindly provided by H. Oberleithner of the University Muenster, Germany. The ratio of RBC sedimentation rates in high over low sodium solutions gives an estimate of individual ESS. ESS ratios <4 were defined as low SS, >4 as high SS, respectively.

A two-sided significance level of *p* < 0.05 was applied to all calculations. Data were analyzed by IBM-SPSS^®^ Statistics 20. Graphics were designed by SigmaPlot^®^ 11.0.

## Results

From the years 2010–2015, data of 88 consecutive patients collected at one single center were included for analysis. Table 1 lists the baseline characteristics. Median age was 59.7 ± 10.8. The average office BP at inclusion was 168.5/91.1 ± 18.2/16.1 mmHg, the 24-h ABP was 151.7/86.8 ± 15.2/11.1 mmHg. The patients received at baseline a median of 5.6 ± 1.4 antihypertensive drugs. In a subgroup of *n* = 37 patients, a SBT before RDN therapy was performed. Using this *in vitro* assay the patients were divided in SS and salt-resistant (SR).

**Table 1:** Main baseline characteristics of treated patients and subgroup differentiation salt-sensitive (SS) vs. salt-resistant (SR) using the in vitro salt blood test.

	All included pat. (*n* = 88)	Subgroup SS (*n* = 15)	Subgroup SR (*n* = 22)	SS vs. SR
Age (years)	59.7 ± 10.8	57.3 ± 11	63.7 ± 9.2	*p* = 0.06
<65 years	58 (65.9%)	12 (80.0%)	11 (50.0%)	*p* = 0.1
Sex m/f	56/32	9/6	14/8	*p* = 0.9
BMI (kg/m^2^)	30.8 ± 5.3	31.4 ± 5.5	31.4 ± 4.6	*p* = 0.9
Antihypertensives	5.6 ± 1.4	5.7 ± 1.3	5.5 ± 1.9	*p* = 0.8
Diabetes (all type)	33 (37.5%)	7 (46.7%)	8 (36.4%)	*p* = 0.6
Obstructive sleep apnea	56 (63.6%)	9 (60.0%)	19 (86.4%)	*p* = 0.2
Responder	57 (64.8%)	9 (60.0%)	13 (59.1%)	*p* = 0.9
Office SBP	168.5 ± 18.2	171.7 ± 15.4	167.5 ± 16.6	*p* = 0.4
Office DBP	91.1 ± 16.1	91.8 ± 14.8	90.5 ± 15.4	*p* = 0.8
24-h SBP	151.7 ± 15.2	156.1 ± 19.8	157 ± 14.9	*p* = 0.9
24-h DBP	86.8 ± 11.1	88.9 ± 8.3	85.4 ± 12.8	*p* = 0.4
Creatinine (mg/dl)	1.1 ± 1.3	1.0 ± 0.4	1.0 ± 0.3	*p* = 0.8
GFR CKD-EPI (ml/min/1.73 m^2^)	79.2 ± 23.5	79.7 ± 25.9	74.5 ± 19.2	*p* = 0.5
Cystatin C (mg/l)	1.0 ± 0.6	1.1 ± 0.5	1.0 ± 0.3	*p* = 0.8
eGFR (ml/min/1.73m^2^)	93.3 ± 36.2	81.3 ± 30.3	86.6 ± 24.4	*p* = 0.6
Urea (mg/dl)	40.4 ± 19	38.6 ± 11.9	38 ± 15.6	*p* = 0.9
Microalbuminuria (mg/dl)	167.7 ± 439.4	201.3 ± 347.2	98.6 ± 240.7	*p* = 0.3
Sodium (mmol/l)	141.9 ± 3	141.9 ± 2.5	141.3 ± 2.6	*p* = 0.5
Potassium (mmol/l)	4.0 ± 0.5	4.0 ± 0.4	4.0 ± 0.4	*p* = 0.7
Hemoglobin (g/dl)	14.0 ± 1.3	13.9 ± 0.9	14.2 ± 1.3	*p* = 0.5
Glucose (mg/dl)	130.9 ± 38.6	133.6 ± 41.9	143 ± 55	*p* = 0.6
HbA1c (%)	6.2 ± 1.1	6.5 ± 1.6	6.4 ± 1.5	*p* = 0.8
Insulin (mU/l)	24.1 ± 27.7	32.0 ± 52	22.1 ± 14.9	*p* = 0.4
c-Peptide (μg/l)	4.2 ± 2.6	4.6 ± 3.6	4.1 ± 1.9	*p* = 0.6
Aldosterone (pg/ml)	102.7 ± 66.7	77 ± 54.1	114.1 ± 74.8	*p* = 0.1
Renin (pg/ml)	63 ± 200.4	22.8 ± 32.2	14.9 ± 28.5	*p* = 0.5
Metanephrine (ng/l)	47.4 ± 23.3	38.8 ± 14.2	42.5 ± 15.4	*p* = 0.5
Normetanephrine (ng/l)	79.2 ± 33.7	73.0 ± 20.1	64.8 ± 25.3	*p* = 0.3
Cholesterol (mg/dl)	199 ± 40.6	195.5 ± 27	194.9 ± 41.9	*p* = 0.9
HDL (mg/dl)	49.8 ± 15.1	44.8 ± 14.5	49.0 ± 13.5	*p* = 0.4
LDL (mg/dl)	128.8 ± 38.5	127.1 ± 26.7	128.1 ± 51.8	*p* = 0.9
NT-proBNP (pg/ml)	307 ± 504.6	586.2 ± 960.1	266.5 ± 447.6	*p* = 0.2

*Data are *n* (%) or mean (SD). BMI, body–mass index; SBP, systolic blood pressure; DBP, diastolic blood pressure; GFR, glomerular filtration rate; eGFR, estimated GFR; HbA1c, glycosylated hemoglobin; HDL, high density lipoprotein; LDL, low density lipoprotein; NT-proBNP, N-terminal pro-brain natriuretic peptide*.

**Figure 1:**
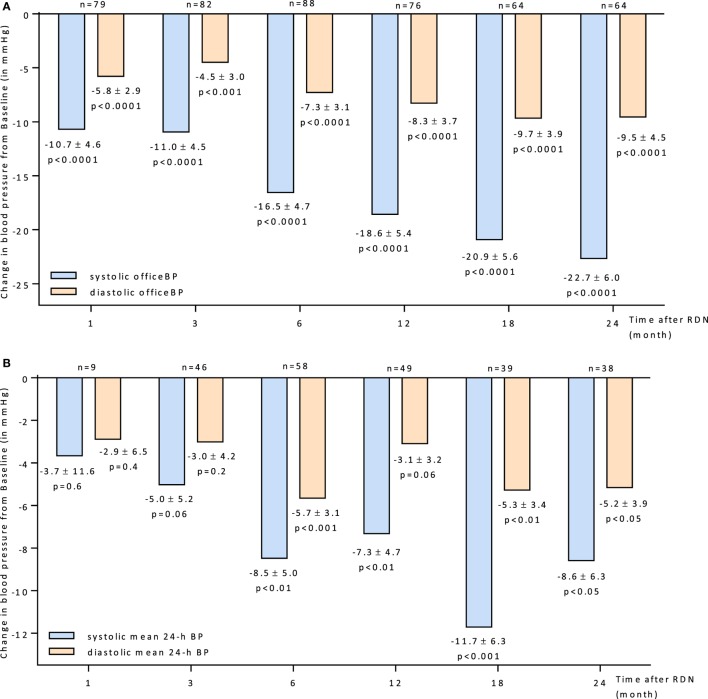
**(A)** Changes up to 24 months in systolic and diastolic office BP for all patients 95% CIs and unadjusted *p* values shown. **(B)** Changes up to 24 months in systolic and diastolic mean 24-h BP for all patients 95% CIs and unadjusted *p* values shown. Abbreviations: RDN, renal denervation; BP, blood pressure.

The overall BP reduction after RDN is shown in Figures 1A,B. There was a significantly reduction of BP observed at all follow-up time points according to the office (Figure 1A) and 24 h BP (Figure 1B) readings. Six months after RDN the Office BP and the 24 h mean BP dropped by −16.5/−7.3 mmHg and −8.5/−5.7 mmHg, respectively.

The overall responder rate after RDN was calculated to 64.7%. A positive BP response to RDN was defined as follows: systolic office BP reduction of at least 10 mmHg after 6 months (15). There were no significant differences between the *n* = 57 responders and the *n* = 31 non-responders in terms of their baseline characteristics except the following: in the responder group, there were more patients <65 years (72% responder vs. 58% non-responder; *p* < 0.05) and their systolic office BP was higher (171.7 ± 18.1 vs. 162.5 ± 17.2; *p* < 0.05).

**Figure 2:**
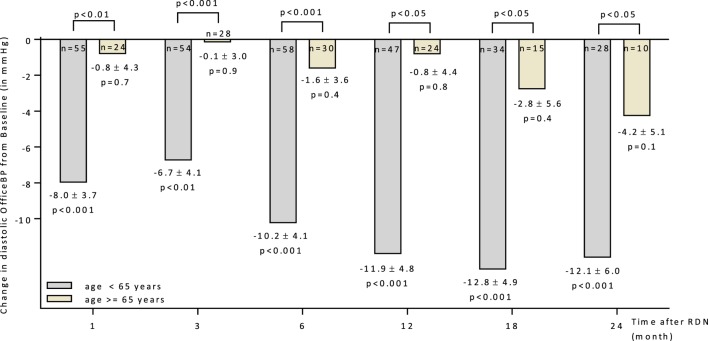
Changes up to 24 months in diastolic office blood pressure for patients age above and below 65 years. 95% CIs and unadjusted *p* values shown. Abbreviations: RDN, renal denervation; OfficeBP, office blood pressure.

A subgroup analysis separating patients below and above 65 years could demonstrate that younger patients respond better to RDN (Figure 2). Six months after RDN, the diastolic office BP dropped significantly in the patients younger than 65 years but not in the group of patients >65 years of age (−10.2 ± 4.1 mmHg vs. −1.6 ± 3.6 mmHg in <65 vs. >65 years).

For impending analyses, the patients were allocated in SS and non-salt-sensitive (NSS) according to their salt sensitivity testing results. As shown in Table 1, no significant differences including age were detected between both groups. Subgroup allocation was done according to the SBT groups. ESS ratios <4 were defined as low SS, >4 as high SS. Accordingly, the ESS ratios were significantly different between both groups. The mean ESS ratio in the SS group was 6.5 ± 1.6 vs. 3.0 ± 0.7 in the SR group (*p* < 0.01).

### Response to RDN According Salt Sensitivity

**Figure 3:**
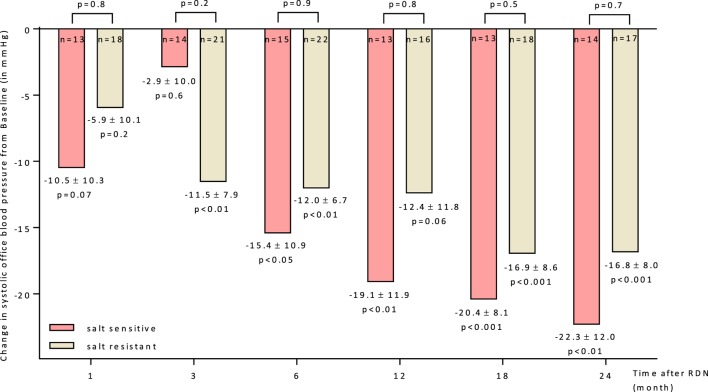
Changes up to 24 months in systolic office blood pressure for salt-sensitive and salt-resistant groups. 95% CIs and unadjusted *p* values shown. Abbreviation: RDN, renal denervation.

The BP reducing effect of RDN did not differ significantly between both groups. The responder rates (SBP reduction ≥ 10 mmHg 6 months after RDN) were 60.0 and 59.1% in SS and SR patients, respectively. The progression in office BP after RDN within the follow-up of 24 months was similar in both groups (Figure 3). There was no statistical difference in the BP lowering effect responding to RND between SS and SR patients at month 6 (28 ± 16 vs. 23 ± 9 mmHg). Successful ABPM readings at month 0 and 6 were available for *n* = 24 patients. There were no differences in baseline (Table 1) and follow-up mean values apparent (24 h mean SS: month 6: 147/83 ± 6/4 mmHg; SR: month 6: 144/77 ± 6/3 mmHg).

**Figure 4:**
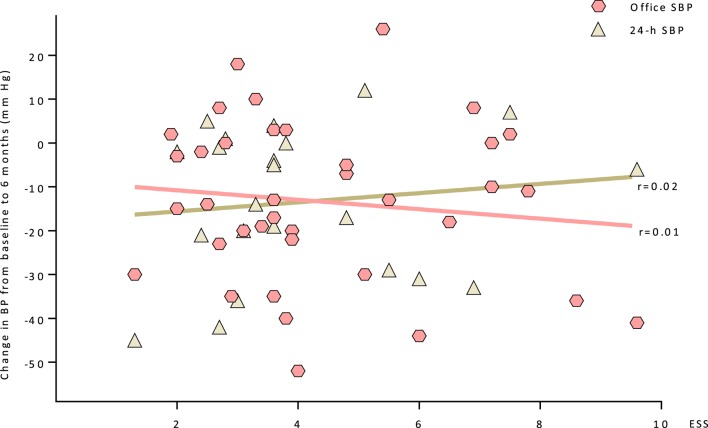
A correlation between the salt sensitivity (ESS) and the BP response to RDN at the 6 months follow-up is shown (systolic office and 24 h BP measurements). Abbreviations: ESS, erythrocyte sodium sensitivity; SBP, systolic blood pressure.

There is no apparent correlation between the salt sensitivity according ESS-Testing and the BP response to RDN as shown by systolic office and 24 h BP measurements at the 6 months follow-up (Figure 4).

In Table 2 the data on urinary sodium excretion in both SR/SS and responder/non-responder groups are shown. There are no difference between both groups and no difference within the groups, respectively.

**Table 2:** The urinary sodium excretion in salt-resistant (SR) and salt-sensitive (SS) patients as well as responder and non-responder groups are shown.

	SR	SS		Responder	Non-Responder	
Baseline (*n* = 28)	107.7 ± 56.2	115.9 ± 32.6	*p* = 0.3	106.8 ± 45.7	90.2 ± 41.4	*p* = 0.6
1 Month (*n* = 27)	101.5 ± 45.9	80.0 ± 29.5	*p* = 0.2	96.3 ± 41.1	98.5 ± 40.9	*p* = 0.9
3 Month (*n* = 27)	88.6 ± 46.3	97.5 ± 42.5	*p* = 0.7	92.7 ± 43.8	101.2 ± 46.5	*p* = 0.7
6 Month (*n* = 20)	94.7 ± 45.8	131.7 ± 46.5	*p* = 0.1	103.6 ± 42.4	103.6 ± 42.9	*p* = 1.0
12 Month (*n* = 26)	97.1 ± 33.5	108.1 ± 39.9	*p* = 0.5	101.1 ± 41.1	101.6 ± 37.3	*p* = 1.0
18 Month (*n* = 14)	91.7 ± 20.6	130.0 ± 73.8	*p* = 0.4	91.6 ± 29.9	127.2 ± 43.2	*p* = 0.7
24 Month (*n* = 9)	107.3 ± 31.1	121.3 ± 58.7	*p* = 0.7	115.4 ± 44.6	103.3 ± 34.9	*p* = 0.8

*No statistical differences between the groups were apparent (Wilcoxon- and Mann–Whitney Test)*.

*Unit: mmol/l; values in mean ± SD*.

## Discussion

Renal denervation just recently came back into focus, since the latest randomized, sham-controlled trial was able to show a benefit of the procedure (6). Numerous preclinical studies showed, that sympathetic nerve activity plays a major role in the development of hypertension. In human clinical trials there are numerous factors, like inhomogeneous patient characteristics, adherence problems, procedural weakness and other confounders that can dilute and even eliminate the anticipated effect as it happened in the past (4).

In this single center analysis, a considerable proportion of patients benefited from RDN. As also shown in other trials particularly patients with the high baseline BP are more likely to respond (16). The responder rate of 64.7% in this analysis and the overall BP reduction of −17/−7 mmHg in office BP and −8/−6 mmHg in 24 h mean BP accordingly, was comparable to other RDN trials (16–19). In the recently published Austrian Transcatheter National Multicentre Renal Denervation (TREND) Registry, Zweiker and colleagues found very similar results in their 188 RDN patients treated in 14 centers. A reduction of −20/−7 mmHg in office BP and −8/−5 mmHg in 24 h mean BP was described (16). The large Global Symplicity Registry that includes the German GREAT Registry showed in their set of 998 patients treated in 134 centers a reduction of −12 mmHg in office BP 6 months after RDN (ABPM reduction −7 mmHg) (13).

As observed by others, older patients with high central aortic BP or isolated systolic hypertension tend to be less susceptible to RDN (20, 21). Correspondingly, patients at an age >65 years did not respond well to RDN in our study. In older patients with a long standing arterial hypertension resulting in a higher arterial stiffness with an enlarged pulse–pressure (blood pressure amplitude) the role of the sympathetic nervous system in driving the elevated BP seems to be less important.

To find other factors predicting response besides age and BP, salt sensitivity was tested prior RDN for the first time. It was hypothesized, that being SS might have an impact on the response to RDN. The particular effect of renal nerves on the regulation of arterial BP and sodium balance is still not fully understood (22). Various indicators of sympathetic drive can be altered by RDN. Dorr and colleagues found a correlation of BP response to RDN and reduction in the level of the sympathetic cotransmitter Neuropeptide Y (23). Recently, Poss and coworkers were able to demonstrate a positive effect of RDN on sodium excretion (9). Noradrenaline, the most important sympathetic neurotransmitter modulates salt handling and, therefore, regulates BP though different mechanisms.

Thus, it has been shown that stimulation of alpha and beta adrenergic receptors directly activates the thiazide-sensitive NaCl cotransporter leading to sodium reabsorption (1). In addition, sympathetic neurotransmitters increase renal vascular resistance and induce renin release thereby regulating water and salt balance (22, 24). Despite this overwhelming evidence for an important role of renal sympathetic nerve activity on salt handling, the present study demonstrated that salt sensitivity does not seem to influence the BP response to RDN. Moreover, the blood pressure reduction in patients responding to RDN was similar between SS and NSS indicating that salt sensitivity did not affect the outcome after RDN. Therefore, salt sensitivity seems not to be a suitable predictor for successful RDN.

As mentioned in the Section “Introduction,” there seems to be a relation between the salt sensitivity in man and arterial hypertension. However it has to be mentioned, that this pathophysiologic relationship has been as yet not fully investigated in clinical trials with large patient numbers. Therefore, it is unknown whether salt sensitivity measured by the SBT could be an useful predictor for selecting hypertensive patients who will respond to RDN. This should be investigated in further studies. Larger trials confirming the clear correlation between the SBT and the renal sodium handling and excretion or response to sodium loading are missing and need to follow.

The HTN-3 trial subgroup analysis might support this observation. The response to RDN in the rather SS Afro-American population was not more pronounced compared to Caucasians (5).

Renal denervation fails to reduce BP in 20–40% of patients. Auxiliary predictors for response could not be identified yet. The present study suggests that salt sensitivity, measured using the *in vitro* assay SBT, does not influence the response to RDN.

The small sample size of the presented data is certainly a limitation of this analysis. In addition, the *in vitro* assay SBT can only estimate the salt sensitivity of the tested patients. A larger trial with conventional salt loading would be needed for more dependable results. We have to continue to study the underlying mechanism how RDN interacts with BP control in order to identify suitable patients for this therapy. Differentiation between SS and not SS does not seem to be helpful in predicting response to RDN.

## Ethics Statement

Ethic commitee University Duesseldorf Ethic No. 3848.

## Author Contributions

OV conducted the trial and has written the manuscript. OM collected the trial data, performed statistical analyses, and arranged the graphics. LR has written parts of the manuscrips, edited the manuscript. PS collected trial data and performed renal denervation. JS conducted the trial and has written the manuscript.

## Conflict of Interest Statement

The authors declare that the research was conducted in the absence of any commercial or financial relationships that could be construed as a potential conflict of interest.
